# Remote Patient Monitoring Program for COVID-19 Patients Following Hospital Discharge: A Cross-Sectional Study

**DOI:** 10.3389/fdgth.2021.721044

**Published:** 2021-11-08

**Authors:** Khayreddine Bouabida, Kathy Malas, Annie Talbot, Marie-Ève Desrosiers, Frédéric Lavoie, Bertrand Lebouché, Melissa Taguemout, Edmond Rafie, David Lessard, Marie-Pascale Pomey

**Affiliations:** ^1^University of Montreal Hospital Centre de Recherche du Centre Hospitalier Universitaire de Montréal (CRCHUM), Montreal, QC, Canada; ^2^École de Santé publique, Département de Gestion, Université de Montréal, Montreal, QC, Canada; ^3^Excutive Office, Centre Hospitalier Universitaire de Montréal (CHUM), Montreal, QC, Canada; ^4^Département de Recherche, Montreal Cancer Institute, University of Montreal Hospital Centre (CRCHUM), Montreal, QC, Canada; ^5^Innovation Axis, Research Center of the CHUM, Montreal, QC, Canada; ^6^Network Coordination Department, CHUM, Montreal, QC, Canada; ^7^Canadian Institutes of Health Research Strategy for Patient-Oriented Research Mentorship Chair in Innovative Clinical Trials in Human Immunodeficiency Virus (HIV), Montreal, QC, Canada; ^8^Centre for Outcomes Research and Evaluation, McGill University Health Centre Research Institute, Montreal, QC, Canada; ^9^State-of-the-Art Technology and Methods, Montreal, QC, Canada; ^10^Center of Excellence of Patient Partnership and the Public, Montreal, QC, Canada; ^11^Department of Health Management, Evaluation, and Policy, School of Public Health, Université de Montréal, Montreal, QC, Canada

**Keywords:** COVID-19, remote patient monitoring, telehealth, telemonitoring, user experience, evaluation

## Abstract

**Background:** The COVID-19 pandemic created an urgent need to act to reduce the spread of the virus and alleviate congestion from healthcare services, protect healthcare providers, and help them maintain satisfactory quality and safety of care. Remote COVID-19 monitoring platforms emerged as potential solutions.

**Objective:** The purpose of this study was to evaluate the capacity and contribution of two different platforms used to remotely monitor patients with COVID-19 to maintain quality, safety, and patient engagement in care, as well as their acceptability, usefulness, and user-friendliness from the user's perspective. The first platform is focused on telecare phone calls (Telecare-Covid), and the second is a telemonitoring app (CareSimple-Covid).

**Methods:** We performed a cross-sectional study. The data were collected through a phone survey from May to August 2020. Data were analyzed using descriptive statistics and *t*-test analysis. Participants' responses and comments on open-ended questions were analyzed using content analysis to identify certain issues and challenges and potential avenues for improving the platforms.

**Results:** Fifty one patients participated in the study. Eighteen participants used the CareSimple-Covid platform and 33 participants used the Telecare-Covid platform. Overall, the satisfaction rate for quality and safety of care for the two platforms was 80%. Over 88% of the users on each platform considered the platforms' services to be engaging, useful, user-friendly, and appropriate to their needs. The survey identified a few significant differences in users' perceptions of each platform: empathy toward users and the quality and safety of the care received were rated significantly higher on the CareSimple-Covid platform than on the Telecare-Covid platform. Users appreciated four aspects of these telehealth approaches: (1) the ease of access to services and the availability of care team members; (2) the user-friendliness of the platforms; (3) the continuity of care provided, and (4) the wide range of services delivered. Users identified some technical limitations and raised certain issues, such as the importance of maintaining human contact, data security, and confidentiality. Improvement suggestions include promoting access to connected devices; enhancing communications between institutions, healthcare users, and the public on confidentiality and personal data protection standards; and integrating a participatory approach to telehealth platform development and deployment efforts.

**Conclusion:** This study provides preliminary evidence that the two remote monitoring platforms are well-received by users, with very few significant differences between them concerning users' experiences and views. This type of program could be considered for use in a post-pandemic era and for other post-hospitalization clienteles. To maximize efficiency, the areas for improvement and the issues identified should be addressed with a patient-centered approach.

## Introduction

### Background

The coronavirus disease (COVID-19) pandemic has had many tragic effects and has been seriously testing the crisis response capacity of health systems around the world (1, 2)^1^^,^^2^. On March 11, 2020, the World Health Organization (WHO) declared the novel coronavirus outbreak a global pandemic (1). With the absence of effective vaccines and therapies to treat SARS-CoV-2 infections, lockdowns, physical distancing, and quarantine measures were adopted and generalized to minimize the impact and slow the spread of the disease as vaccines were being developed and approved and were proven effective by the end of 2020 (1, 2)^1^^,^^2^.

However, these measures have had negative impacts on healthcare users (1–3)^1^, including difficulties accessing care, isolation, anxiety, and depression, that have affected patients, their loved ones, and healthcare professionals, and had negative impacts on health outcomes and the quality of care provided (1–3). To counter these effects and maintain a high quality of care, health systems innovated and developed new models of care and intelligent remote patient monitoring (RPM) strategies that employ telehealth platforms (4–11)^1^. As early as the spring of 2020, various interventions using connected platforms were rapidly developed to deal with the virus. Several studies have presented telehealth platforms such as mobile health apps and several telemonitoring connected devices and telecare programs as promising solutions and reliable technological tools (4–11). It has been suggested that with their telemonitoring/telecare capacities and remote monitoring capabilities, telehealth platforms can provide patients with practical and timely access to care (4–11). Telehealth offers asynchronous communication, collecting and tracking data but also obtaining real-time clinical feedback that is well-suited to the remote patient monitoring process (4–12). Moreover, health technology experts and healthcare leaders have suggested that telehealth and RPM platforms can help facilitate continuity of care and provide considerable support for the organization and administration of care services during the current pandemic (4–12).

In this context, the Centre of Network Flow Optimization (CNFO) at the Hospital Center of the Université de Montrèal (CHUM), a major public University hospital in Canada, has developed and adapted two technological platforms to remotely monitor patients with COVID-19 following a hospital visit or discharge^3^^,^^4^.

The first platform in this program is the TELECARE calls platform, which we will call Telecare-Covid in this paper. The second platform is a telemonitoring app called the CARESIMPLE Platform, which we will call CareSimple-Covid. Depending on their wishes and preferences, patients with COVID-19 have a choice when they are discharged: to be remotely monitored through the services of either the Telecare-Covid calls platform or the CareSimple-Covid app program. Patients can also choose to use both if they wish. The Telecare-Covid platform is a clinical follow-up incoming calls system with phone lines available 24/7 and dedicated to receiving calls from COVID-19 patients. Patients can discuss their clinical symptoms directly with a nurse, who will process and assess the clinical information. The CareSimple-Covid platform is a telemonitoring app downloadable on Android and iOS smartphone and tablet systems. Over the CareSimple-Covid platform, patients can enter and submit data on their symptoms and clinical information twice daily. The symptoms are then gathered, processed, and assessed automatically by the system. If the system detects a deterioration in the patient's health, a nurse will be notified directly, call the patient to check their symptoms, and further evaluate the situation with a physician and members of the care team. For both platforms, if the situation requires an urgent intervention, a transfer to the hospital will be offered by the call center staff. The staff is dedicated to the platform and consists of nurses, residents, and physicians accessible by phone and working 24/7^3^^,^^4^. Before referring patients to the remote monitoring platforms, selection criteria are considered, including the health status of patients and the progress of their COVID-19 disease, their ability and motivation to use the platforms, and their preferences. Based on these criteria, CNFO nurses managing the remote monitoring program will identify potential users among the COVID-19 patients discharged from the hospital. Then a CNFO nurse will present and explain to the patient how the two platforms work, and if a patient expresses an interest in using one of the platforms, the care team provides the necessary tools and information on how to use it. A technical support team available 5 days per week has been also included in the program to help resolve any technical or IT issues on either platform.

Although the two remote monitoring platforms operate in different ways, they were developed and adapted to achieve the same goals of providing (1) a safer return home for patients who are medically stabilized but at risk of decompensation by guaranteeing regular clinical follow-up and continuous remote monitoring for 14 days; (2) emotional support to reduce isolation and anxiety in patients by connecting them to clinical teams; (3) a medical safety net to reduce the risk of SARS-CoV-2 infections within care services; (4) improved workflows and reduced congestion in care services, which have been exacerbated by the pandemic, through better control of unnecessary visits to care services and facilities; and (5) eventually, continued good quality and safety of care.

### Objectives

The objectives of this study are to (1) evaluate the user-friendliness of Telecare-Covid and CareSimple-Covid and through patient self-report how, they can provide quality, safe, and engaging care to patients; (2) identify factors that lead patients to choose one platform over another; (3) explore patients' perceptions of the added value provided by the platforms; and (4) identify any required improvements in how the platforms are used, from the patient's perspective.

## Methods

### Study Design

A cross-sectional study was conducted using a survey of COVID-19 patients who were remotely monitored on the two platforms (13–18). This study received ethical approval from the Research Ethics Committee of the Université de Montréal Hospital Research Center (CRCHUM) (CER-CHUM: 20.040).

To achieve the study's objectives, we used three validated questionnaires that we adapted to the COVID-19 context to evaluated patients' perceptions on the following dimensions (19–21):

1. Quality and safety of care (access, safety, relevance, timeliness, etc.) (19);
2. Patient engagement and partnership (participation, collaboration, trust, empathy, recognition, relationship with the care team, etc.) (20);
3. The utilization capacity of the telehealth platforms (user-friendliness, usefulness, problems encountered, etc.) (21); and
4. The sociodemographic characteristics of the COVID-19 patients who used the two platforms (20).

A validated questionnaire of 20 questions grouped in 5 sections, including questions rated on a 5-point Likert scale (1—strongly disagree to 5—strongly agree), multiple-choice questions, and a general comments section, was administrated to the participants (Table 1). In the general comments section, participants were asked to share their thoughts on their experience with the platform. The general comments helped us identify what participants did and did not appreciate when using the platform. This also allowed us to identify some important issues and factors that lead patients to choose one platform over the other. Participants provided suggestions for improving the platforms and improving user experience in the general comments section.

**Table 1:** Dimensions and elements examined with the questionnaire.

**Section/dimension**	**Questionnaire items/attributes**
Demographic characteristics of users (20)	Gender^a^
	Age^a^
	Geographic area^a^
	Living situation (living alone or with another person)^a^
Perceptions of the quality and safety of care (19)	Availability and access to a member of the care team at all times^b^
	Pertinence and frequency of the care received ^b^
	Consideration of the psychological impacts of the care received from the care team^b^
	Support and consideration provided to the patient by the care team^b^
	Satisfaction with the quality and safety of the care received through the platform^b^
Perceptions of patient engagement in care and the relationship with the care team (20)	Information received on health status and care^b^
	Information given and communicated to healthcare teams on health status^b^
	Engagement in care and partnership with the care team^b^
	Patient participation in the decision making related to care^b^
	Decision making according to the patient's needs and preferences^b^
	Bond of trust with the health care team^b^
	Importance of the information received and shared between the care team and the patient^b^
	Empathy expressed between the patient and the healthcare team^b^
	Recognition of the patient's experience with the disease by the healthcare team^b^
Perceptions of utilization capacity (usefulness, user-friendliness, problems) (21)	Services offered by the platform are useful and meet the needs of users^b^
	User-friendliness and problems encountered while using the platform^a^
General comments (optional)	Additional comments and suggestions on the general utilization experience (improvements, issues, concerns, etc.)^c^

a *Multiple choice question*.

b *Likert scale question: (1-strongly disagree to 5-strongly agree)*.

c *Open question (Additional Comment)*.

Note, that the adaptation brought to the questionnaire is regarding two elements. The first one is linking the questions to the covid 19 contexts. The original questionnaire suggests when administrating the items to start the question by linking it to the disease or the clinical problem that motivates the patient to use the technological platform. e.g., In the context of the Covid-19 health crisis, “The platform care simple/telecare responded well to my needs or patient's needs?” also we added an open question in the last section of the questionnaire for general comments.

The second is on the demographic information. In fact, the demographic information collected through our question has been limited to age gender, and household composition, and geographical region. Although we wanted to collect the socioeconomic status, education, ethnic background as it was done in the original questionnaire, from our experience during the testing phase for the acceptability of the survey prior to the official questionnaire administration, we learned that 80% of respondents left the space blank when it came to those demographic questions and that is why we decided to not include them in the demographics section.

### Recruitment

The selection criteria used to recruit the participants were: all patients infected with SARS-COV2 registered on the CNFO remote monitoring program who used at least one of the two platforms for 14 days following a hospital discharge from April to June 2020.

### Data Collection

The data were collected remotely from May to August 2020. Data were collected by a team of three trained on good clinical research practices and on conducting interviews and administrating questionnaires. The data collection team included one Ph.D. Candidate and two students in the second year of a medical program (MD). After they have given their consent, participants were invited to complete the questionnaire through a scheduled phone call with the data collection members of the research team. Then the data collected were entered and recorded in CRCHUM's secure “REDCap” computer system^5^, which was designed specifically for surveys and quantitative data collection and processing.

### Data Analysis

We used a quantitative design approach for the study, with only general comments being processed from a qualitative perspective (15). Data analysis was performed concurrently with data collection to allow for an iterative approach (15, 18).

We conducted a descriptive and *t*-test statistical analysis of the data collected using SPSS (Statistical Package for the Social Sciences) information processing software (13–18). We used descriptive statistics to describe, in a summative and complete way, the data on the four evaluated dimensions and to identify positive or negative trends in the results (13–18). Through this analysis, we arrived at a description of some central trends (mean, median, standard deviation) in participants' views. To identify significant differences in their views on the user experience with the two platforms, we performed a *t*-test analysis. The general comments collected in the last section of the questionnaire were transcribed verbatim and analyzed through content analysis using QDA Miner qualitative data analysis software (15, 17, 18).

The data analysis was performed and reviewed by all members of the research team to ensure a high level of validity using an inter-researcher triangulation strategy (13–18). Interim reports and presentations were also communicated to the patients, participants, and actors involved in the platform's development, deployment, and use (patients, clinicians, managers, volunteers, etc.). These exchanges helped strengthen the validity of the analysis to help us compare our interpretations with those of the participants.

## Results

Table 2 presents the number of participants and participation rates in the study.

**Table 2:** Number of users, rate of use, and participation.

	**Platform users**			**Survey participants**		
	**Total**	**Telecare-Covid**	**CareSimple-Covid**	**Total**	**Telecare-Covid**	**CareSimple-Covid**
Number (*n*)	85	65	20	51	33	18
Rate (%)	100	76	24	60	53	90

A total of 85 patients with COVID-19 diagnosed or hospitalized at CHUM from April to June 2020 agreed to register and use the remote monitoring program proposed by CNFO upon discharge from the hospital or after a hospital visit. Sixty-five patients (76%) used the Telecare-Covid platform and 20 patients (24%) used the CareSimple-Covid platform.

In total, 51 patients (participation rate of 60%) participated in the study: 18 participants used the telemonitoring app CareSimple-Covid (participation rate of 90%), and 33 participants used the Telecare-Covid platform (participation rate of 53%) (see Table 2).

### Demographics

The average age of the participants was 52 years (standard deviation, SD = 13.5) and varied from 24 to 90 years old. Twenty-eight participants were female (55%) and 23 were male (45%). The majority of the users live in Montreal (76%) with at least one other person (73%) (see Table 3). Comparing the demographic characteristics of the users of the two platforms, even though there is a numerical difference, no statically significant difference was found based on the *t*-test (*p*-value < 0.05) for independent samples. We found no significant differences in the distributions of users' demographic characteristics between the two platforms (confidence level of 95%). Thus, the sample of those who participated in the study (51 patients) is demographically representative of the larger group of patients who used the platforms (85 patients) (Table 3).

**Table 3:** Users' demographics.

**Characteristics**	**Total**	**CareSimple-Covid**	**Telecare-Covid**	***P*-value**
Patient gender *n* (%)	*N* = 51 (%)	*N* = 18 (%)	*N* = 33 (%)	
Female	28 (55)	9 (50)	19 (56)	^a^
Male	23 (45)	9 (50)	14 (44)	^a^
Age groups *n* (%)	*N* = 51 (%)	*N* = 18 (%)	*N* = 33 (%)	
20–39	10 (20)	4 (22)	6 (18)	^a^
40–59	28 (55)	8 (44)	20 (61)	^a^
60 or +	13 (25)	6 (33)	7 (21)	^a^
Mean	52	52	52	–
Median	52	56	50	–
Minimum	24	35	24	–
Maximum	90	65	90	–
SD	13.5	10.9	15.4	–
What region do you live in *n* (%)	*N* = 51 (%)	*N* = 18 (%)	*N* = 33 (%)	
Montreal	39 (76)	13 (72)	27 (89)	^a^
Lanaudière	6 (13)	4 (22)	2 (4)	^a^
Laval	3 (6)	1 (6)	2 (4)	^a^
Montérégie	2 (4)	0 (0)	2 (4)	–
Composition of your household *n* (%)	*N* = 51 (%)	*N* = 18 (%)	*N* = 33 (%)	
I live alone	14 (27)	4 (22)	10 (30)	^a^
I live with someone	37 (72)	14 (77)	23 (70)	^a^

a *, Non-signifiant p-values*.

### Perceptions of the Quality and Safety of Care

**Figure 1:**
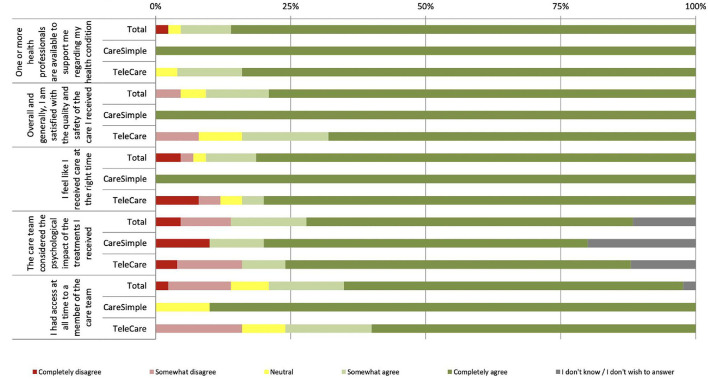
Perception of the quality and safety of care.

More than 80% of the participants completely agreed that they were very satisfied with the quality and safety of the care provided on the two platforms (Figure 1). Overall, participants were satisfied with the quality and safety of the care received through both platforms (mean, *M* = 4.65/5, standard deviation, SD = 0.78). The majority of participants agreed that: they received care promptly on both platforms (*M* = 4; 60/5, SD = 1.00); they had access to a member of the care team at all times (*M* = 4.26/5; SD = 1.17); and medical staff was available to help them deal with their health status (*M* = 4.77/5; SD = 0.72). They also reported, for both platforms, that the care team considered the impact of the provided treatments and services on their psychological state (*M* = 4.32/5, SD = 1.23) (see Table 4).

**Table 4:** Mean and SD relative to perceptions of quality and safety of care (overall and for each platform separately).

**Attributes**	**Total**		**CareSimple-Covid**		**Telecare-Covid**		***p*-value**
	**Mean**	**SD**	**Mean**	**SD**	**Mean**	**SD**	
One or more health professionals are available to support me regarding my health status	4.8	0.7	5	0	4.8	0.5	^a^
Overall and generally, I am satisfied with the quality and safety of the care I received	4.7	0.8	5	0	4.4	1.0	0.0^*^
I feel like I received care at the right time	4.6	1.0	5	0	4.4	1.3	0.0^*^
The care team considered the psychological impact of the treatments I received	4.3	1.2	4.7	1.4	4.5	1.3	^a^
I had access at all times to a member of the care team	4.3	1.2	4.8	0.6	4.2	1.2	^a^

a *, Non-significant p-values*.

* *Statistically significant difference (p < 0.05)*.

Besides the overall response rate, when comparing the participants' mean responses for each platform, the *t*-test for independent samples (*p*-value < 0.05) found statistically significant differences. For two items, “Overall I am satisfied with the quality and safety of the care I received” and “I feel like I received care at the right time,” the mean responses were significantly higher for the CareSimple-Covid platform than for the Telecare-Covid platform (Table 4).

### Perceptions of Engagement in Care and the Relationship With the Medical Team

**Figure 2:**
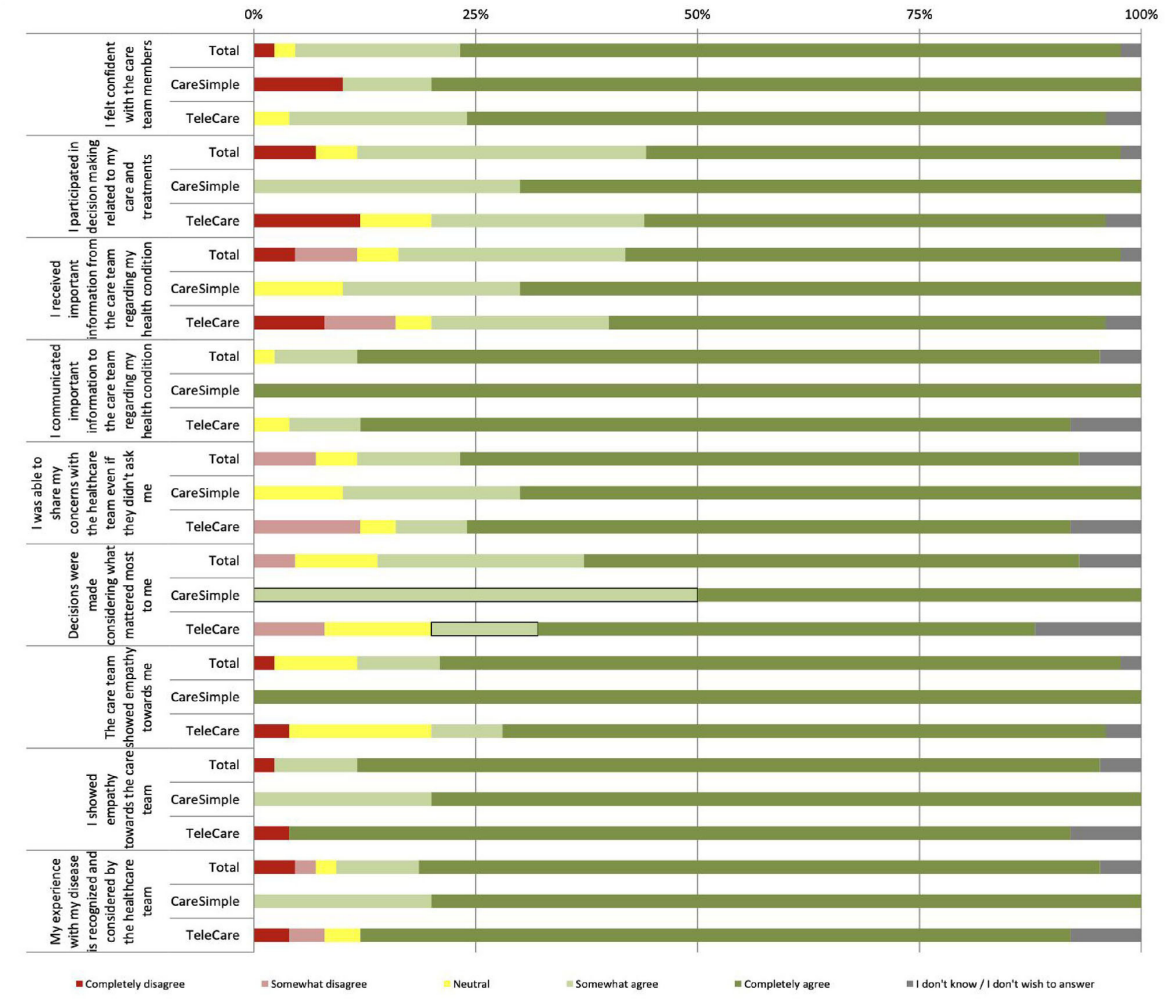
Perception of engagement in care and the relationship with the care team.

Engagement in care and the relationship with the medical team were also very well-rated by the participants (Figure 2). “I gave important information about my condition or my care to the care team” is the attribute that received the highest rating, at 84%, and with no participant disagreeing (Figure 2). Overall, participants reported feeling confident in the care team on both of the platforms (*M* = 4.67/5, SD = 0.75) and participated in the decision making related to their care (*M* = 4.29/5, SD = 1.09). They believe that through the two platforms, they were able to share important information on their health status (*M* = 4.85/5, ET = 0.42) and they also received important information on their health status and the treatments provided (*M* = 4.24/5, SD = 1.14). Concerning the rest of the attributes of this dimension, they all had a mean >4.24 and standard deviation of 0.90–1.02 (Table 5).

**Table 5:** Mean and SD of patient perceptions of engagement in care and the medical team (overall and specific to each platform).

**Attributes**	**Total**		**CareSimple-Covid**		**Telecare-Covid**		***p*-value**
	**Mean**	**SD**	**Mean**	**SD**	**Mean**	**SD**	
I felt confident with the care team members	4.7	0.8	4.5	1.3	4.8	0.6	^a^
I participated in decision making related to my care and treatments	4.3	1.1	4.7	0.5	4.2	1.4	^a^
I received important information from the care team regarding my health status	4.2	1.1	4.6	0.7	4.2	1.4	^a^
I communicated important information to the care team regarding my health status	4.9	0.4	5	0	4.9	0.6	^a^
I was able to share my concerns with the healthcare team even if they didn't ask me	4.6	0.9	4.6	0.7	4.6	1.1	^a^
Decisions were made considering what mattered most to me	4.4	0.9	4.5	0.5	4.5	1.1	^a^
The care team showed empathy toward me	4.6	0.9	5	0	4.5	1.1	0.0^*^
I showed empathy toward the care team	4.8	0.7	4.8	0.8	4.9	0.9	^a^
My experience with my disease is recognized and considered by the healthcare team	4.6	1.0	4.8	0.4	4.7	1.1	^a^

a *, Non-significant p- values*.

* *Statistically significant difference (p < 0.05)*.

When comparing the participants' mean responses for each platform, only one attribute showed statistically significant differences based on the *t*-test for independent samples (*p*-value < 0.05). The mean response for the attribute “The care team showed empathy toward me” was significantly higher for the CareSimple-Covid platform than for the Telecare-Covid platform (Table 5).

### Perception of the Usefulness and User-Friendliness of the Platform

**Figure 3:**
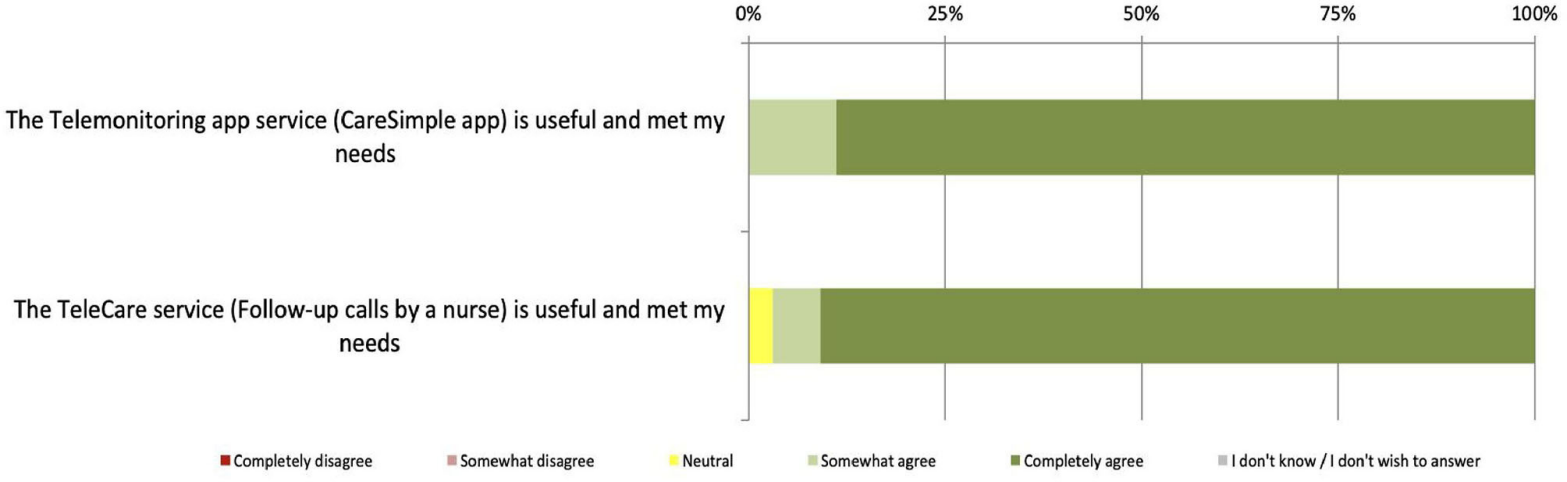
Perception of the usefulness and usability of the platform.

**Figure 4:**
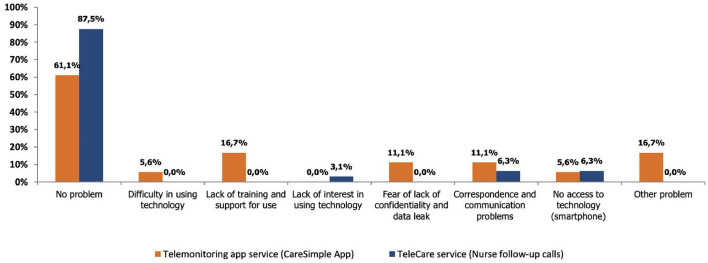
Types of problems encountered using the platform.

Overall, the evaluation of this dimension shows that 91% of participants who used the Telecare-Covid platform and 89% of those who used the CareSimple-Covid platform felt that the services they offer are useful and responded to their needs (see Figure 3). Moreover, 87% of the users of the Telecare-Covid platform said that no problem was encountered while using it, while this was reported by only 61% of the participants who used the CareSimple-Covid platform. The main problems encountered include difficulties using the technology and a lack of training (17%), fears over confidentiality (11%), and difficulties accessing the connected devices (smartphones or tablets, etc.) (6%) (see Figure 4).

Besides the overall response rate, we did not identify any statistically significant differences through the *t*-test on independent samples (*p*-value < 0.05) when comparing the type of problems encountered on each platform (Table 6).

**Table 6:** *T*-test results and comparison of the types of problems encountered in using the two platforms (no significant statistical difference found).

**Problem type**	***p*-value**
No problem	^a^
Difficulty in using technology	–
Lack of training and support for use	–
Lack of interest in using technology	–
Fear of lack of confidentiality and data leak	–
Correspondence and communication problems	^a^
No access to technology (smartphone)	^a^
Other problem	–

a *, Non-significant p-values*.

*(-) means no comparison was possible (i.e., this category is not used in the comparisons because the proportion is equal to zero)*.

### Results From the General Comments

Forty-three participants completed the general comments section (the optional section) of the survey. Of this group, 15 used the CareSimple-Covid platform and 27 used the Telecare-Covid platform. An analysis of the content of the comments allowed us to identify what the patients liked about using these platforms, what needs improvement, and what were their concerns or issues.

### What Was Appreciated?

For both the CareSimple-Covid platform and the Telecare-Covid platform, we identified four main features that were highly appreciated by the majority of participants. First, they liked the ease of access to services in general but, more specifically, the accessibility and availability of the care team. Second, they appreciated the continuity of care and clinical monitoring from the hospital to the home. According to some participants, having the changes in their health status monitored as a continuous process, even if this was done remotely, helped them better manage their concerns about any deterioration in their health once they had returned home. Third, they appreciated the practicality and user-friendliness of the two platforms; in particular, participants noted the dynamism of the CareSimple-Covid platform. Fourth, they liked the diverse range of services offered on the two platforms. Some participants, and especially those who live alone, reported that the psychological help received through the remote monitoring platforms reassured them and helped reduce feelings of isolation and anxiety due to the collateral effects of the quarantine. For example, the participants who used CareSimple-Covid felt that being monitored in this way has a favorable psychological impact because a CNFO nurse would assess and reassure the patient if the patient showed psychological distress through the platform.

### What Needs Improvement?

Although the feedback and comments were generally positive, some participants nevertheless identified areas in which actions should be taken, knowing that these are roughly the same areas presented above in Figure 4. A small number of participants who used the CareSimple-Covid platform emphasized their difficulties accessing or owning connected devices (smartphones, tablets, etc.) and a lack of training in their use. Individuals with chronic health problems and/or a particular medical history spoke of wanting platform services that would be better suited to their realities and clinical profiles. Lastly, some participants requested faster and more responsive services in terms of their communications and correspondence and the remote interaction process with the care team over both platforms.

### What Issues Were Raised?

The general comments and feedback also allowed us to identify certain issues with the two platforms. Participants mentioned their concerns over maintaining human contact in care while stressing the importance of the warmth, connection, and interpersonal engagement between patients and care providers. In addition, data security and confidentiality seemed to be another concern for the participants. Some said that they feared a breach of confidentiality in the care relationship and required more clarity and guarantees regarding their data that is transmitted and shared on virtual platforms so that they could feel completely secure.

The following table presents some participants' quotes for each domain identified above (See Table 7).

**Table 7:** Examples of comments related to specific areas of interest for patients.

	**Comments**	
Areas that were appreciated	1	“I had quick access to the nurse through the telecare service”. P17^a^ “The follow-up services over the app were easily accessible and I felt very close to the medical team”. P21^b^
	2	“It's wonderful, the continuity of services, even after hospitalization”. P11 ^a^ “On the CareSimple-Covid app we have the impression of having a doctor or nurse by our side constantly and continuously, which reassured me a lot”. P23 ^b^
	3	“I found the nurse follow-up calls (on the telecare platform) very easy to use.” P34 ^a^ “The concept of the CareSimple-Covid platform is great and the app is very easy to use.” P43 ^b^
	4	“The telecare services were fantastic. It was really beyond my expectations, and I had access to several services including psychological follow-up. I even had access to a psychiatrist through this platform.” P10 ^a^ “On the CareSimple-Covid I had access to a whole team, a nurse, neurologist, and even psychiatric services”. P38^b^
Areas for improvement	1	“The CareSimple-Covid app was on my daughter's phone. I had a lot of trouble using the service, especially since I don't have a smartphone and I wasn't trained on how to use the app. My daughter used to enter my data on her phone”. P39 ^b^
	2	“Sometimes, the response times were long”. P03 ^b^ “The waiting time for my request to be processed after I entered my data was very long.” P24 ^b^
	3	“I received information about my health status and COVID-19 symptoms, but I didn't receive information related to my clinical history and my other health problems”. P27 ^a^ “It would be a good idea to integrate more specific functionalities for monitoring COVID patients with specific clinical profiles, such as those who have undergone surgery, are pregnant, are taking immunosuppressants, etc.”. P15 ^b^
Other issues	1	“I really would have liked the doctor to be closer to the patient, not just on the phone”. P06 ^a^ “The care team was competent, but honestly I believe it lacked human contact. Sometimes I would have liked to speak directly with the nurse or medical team member, rather than using the CareSimple-Covid app.” P16 ^b^
	2	“It is not very comfortable and reassuring to share certain information through a phone call”. P36 ^a^ “I am worried about confidentiality, and I fear a lack of confidentiality and data leaks.” P28 ^b^

a *, Participant who used Telecare-Covid platform*.

b *, Participant who used the CareSimple-Covid platform*.

## Discussion

### Principal Results

In the era of COVID-19, hospitals have become testing grounds for innovation on how to reconnect COVID-19 patients with care teams, while improving patient flow and minimizing healthcare providers' exposure in a serious pandemic and difficult work conditions. We chose a survey approach to study the contribution that can be made by two different platforms designed and adapted for the remote monitoring of patients with COVID-19. From the point of view of the patients, we evaluated the user experience in various aspects on both platforms. Overall, the findings were encouraging, and the dimensions evaluated have demonstrated remarkable levels of appreciation for both platforms. The questionnaire results suggest that, in general, users' perceptions of the quality and safety of care offered on the two remote monitoring platforms were very positive. Therefore, we assume that both platforms have helped maintain a satisfactory level of quality and safety of care provided remotely. Similarly, users' perceptions of their relationship with the care team and their engagement in their care, despite its being offered remotely, were still favorable. Moreover, the majority of the participants on the two platforms affirmed that the remote monitoring services met their needs and indicated that they did not encounter any problems during use, which demonstrates the usefulness and user-friendliness of both platforms. However, one should also note that the survey identified a couple of significant differences in users' perceptions of certain aspects of each platform where empathy toward users and the quality and safety of the care received were significantly higher on the CareSimple-Covid platform than on the Telecare-Covid platform. We assume that this difference is because the CareSimple-Covid platform is a phone and tablet app that can be more interactive where the access and communication with the care team would be quicker and more dynamic than the Telecare-Covid platform that uses an incoming calls system.

The general comments we received corroborate these conclusions, and they have allowed us to identify and better understand the most valued and appreciated aspects of both platforms. The fact that the majority of participants appreciated the ease of access and the proximity of care teams, the continuity of care, the features' user-friendliness, and the many services offered through the platforms illustrates the concrete and undeniable positive contribution made by the two remote monitoring platforms.

Turning to our interpretation of the results for each platform, although they use two different approaches to remote monitoring, the results show significant differences in participants' perceptions of each platform based on the dimensions assessed. There are nevertheless a few differences in users' perceptions of certain aspects of each platform. More specifically, the results suggest that the users of the CareSimple-Covid platform had slightly better perceptions of the quality and safety of their care, as well as the engagement in care and their relationship with the care team. In contrast, the users of the Telecare-Covid platform had slightly better perceptions of its usefulness and user-friendliness. Furthermore, the participants who used the Telecare-Covid platform reported fewer problems compared to participants who used the CareSimple-Covid platform.

Although the feedback received on the experience of using the two platforms' services was generally positive and favorable, some areas for improvement were mentioned, such as training and access to connected devices as well as the need to customize the platforms further with clinical profiles. Above all, several social acceptability concerns need to be addressed. The first is that some participants mentioned the importance of maintaining human contact when providing care. Second, and despite the elaborate regulatory system approved by both of the institutions (CHUM and CRCHUM) regarding maintaining the confidentiality of data on patients, the issue of confidentiality and data leaks remains a concern to a small number of participants. CHUM and the two platform teams fully complied with the security and data confidentiality measures, and no incident of this kind was reported or observed. However, some individuals may still express concerns and different points of view on this issue, and this is socially understandable.

In summary, the results of this study highlight the contribution made by the two platforms during the first wave of the pandemic (April, May, and June 2020) in Canada. These results provide new information on how we can use technological platforms to support health systems in the continuity of their services, but also in maintaining the quality and safety of care, even during an extraordinary health event. Finally, it should be noted that the platforms were not initially designed to monitor COVID-19 patients; they were multidisciplinary virtual platforms that existed long before the pandemic. But in order to quickly respond to the need to intervene and support care services and maintain safe care of high quality, it was decided to develop and adapt the existing platforms, within a very short timeframe, for remote monitoring of COVID-19 patients. Therefore, in addition to the encouraging results that we recorded, we would like to highlight the success of the decision-making and technical transformation process that allowed us to better exploit the two platforms and quickly respond to urgent needs. This paper provides a sense of the effective collaboration achieved between the CareSimple and Telecare teams and the leaders of CHUM and CRCHUM and the considerable effort invested in this program, which could be considered a good model.

### Suggestions for Improvement

Regarding potential improvements to technical and practical aspects of the platforms, we suggest (1) promoting access to smartphones, tablets, and other connected devices by offering, for example, smartphone and tablet loan services and formally training patients in how to use the platforms by introducing simplified tutorials or practical videos; (2) developing and enhancing the correspondence mechanism to speed up the communication and exchange process between patients and care teams and make it more responsive; and (3) developing and adapting the platforms' content to the needs of COVID-19 patients with chronic diseases and adding more clinical profiles to the platforms to provide a more specific more customized, and less generic follow-up process.

We believe that the most important area for improvement is not technical or practical in nature but rather related to social acceptability concerns. In fact, the issue of maintaining human contact in care, and the issue of confidentiality and data security appear to be real concerns. Hence, on this particular issue, healthcare institutions could better develop their communications with patients and the public. Patients and healthcare users should be systematically informed that the security and confidentiality of their personal data are fully protected by their health institutions. In addition, institutions could better explain and communicate their regulatory standards and ethical principles to the public in order to reassure them and reduce their concerns and skepticism around the use of technological platforms.

Regarding the issue of maintaining human contact when receiving care, we recommend entering into discussions and consultations with patients, the public, and experts in public health, ethics, technology, and politics to address this issue in a transparent and democratic deliberative process. Furthermore, the integration of the participatory 4P (Precise, Predictive, Personalized, Preventive) approach during the development and deployment of telehealth platforms would be a tremendous asset. The 4P approach would better help care providers and other interested parties make the most informed decisions while offering patients greater understanding and control of their choices on how to be monitored and receive care, whether remotely, virtually, or in-person (22, 23).

Finally, research in this area should be promoted, and studies that focus on these particular issues should be facilitated and supported.

### Comparison With Prior Work

This study contributes modestly to enrich and deepen the knowledge already available in the literature in the field of telehealth and telemonitoring in general, but in particular on the impacts and challenges of using such approaches in an extraordinary context. This study also stresses the importance of the decision-making and leadership process that supported and facilitated the successful development of the technological platforms within only 4 weeks, despite the difficult circumstances caused by the COVID-19 pandemic.

In the literature, we find several studies that suggest the positive impact of the use of telehealth platforms, in particular on the quality and safety of care (4–12). The positive impact on the acceptability, usefulness, and user-friendliness of technological tools and devices used in telehealth platforms has also been demonstrated in several clinical fields, notably in long-term care, mental health, and oncology. Since the beginning of the COVID-19 pandemic, the impacts of telehealth platforms have increasingly been studied, tested, and demonstrated in the clinical context of COVID-19 (24–29). Therefore, our study corroborates the findings of numerous other studies, and especially those related to the two areas highlighted above. However, what is special about our study, and what distinguishes it from other studies in the literature on this particular topic, is the innovative application of patient engagement and the partnership with the care team that we have assessed – no other study has evaluated this dimension of patient engagement and partnership with the care team through remote monitoring platforms in the context or clinical setting of COVID-19.

Finally, the concerns raised in our study over social acceptability have often been highlighted in studies on ethics and telehealth, whether or not they were in the context of COVID-19 (30–35).

### Strengths and Limitations

This study has certain advantages. Several stakeholders, researchers, and experts in the field either supervised or were involved in the study. Our intervention has been rigorously and promptly developed to cope with the urgent needs of the first wave of the COVID-19 pandemic in Canada. Nevertheless, our study has some limitations. First, it was a single-center study, and our design did not include a control group, i.e., patients with COVID-19 who were not monitored remotely. Studying the views of patients who did not use the remote monitoring platforms would have been highly worthwhile, and this could have lent support to our main findings. In addition, our inclusion criteria provided a wide range of cases with an unknown variety of comorbidity and clinical profiles among the participants. Furthermore, we did not study all the 85 users registered on the two remote monitoring platforms. Consequently, we consider that our full sample size of 51 for the two platforms was relatively average, and as a result, the two sub-samples for the platforms we studied were not equal. The participation rate in the study varied between users of the two platforms (53% for the Telecare platform and 93% for the CareSimple platform). This may have provided an additional source of bias and may limit the generalizability of our findings.

Finally, we could not go deeper to explore and explain in-depth the issues and concerns yielded in the open-ended questions because of the quantitative design of our study. This will be considered in-depth in our upcoming study regarding the qualitative evaluation of the two platforms. Also, we think that the integration of socio-economical and ethnic information in the demographical section could have been very interesting and beneficial for growing insights and focus on equity of access to digital health but, unfortunately, participants were not responsive to these demographic elements and that's why they have not been considered in this study and we will reconsider integrating those demographics information in the upcoming study.

### Perspectives and Implications for Decision-Makers, Healthcare Professionals and Researchers

In light of the feedback provided by patients in this study on the individual preferences and challenges experienced we can appreciate the importance of measuring the patients' views and exploring their perspectives. This can help improves the services provided and better respond to users' aspirations and respecting their choices in an innovative socio-technological process even in abnormal circumstances such as COVID19. This paper's findings contribute to the growing literature and regarding the pros and cons of remote monitoring and recommendations for improvements. We encourage healthcare professionals and researchers, to conjugate their efforts, collaborate together, and to not only focus their research and evaluation on technical or clinical aspects but also on organizational, social, and of course ethical aspects because as we have seen in our study, the ethical and social aspects the acceptability aspects can occupy an important interest among the care users and patients and these aspects should never be neglected.

Finally, our evaluation experience of the two RPM platforms recognizes the importance of the resources and time required to implement and evaluate new technologies. The RPM platforms, are very promising tools and can bring great added value for both health professionals and health users. However, we learned that RPM platforms need multiple resources to be maintained, supported, managed, and even evaluated and studied such as IT, human and financial resources as well as organizational resources. In addition, these programs require the goodwill, support, and involvement of all actors and stakeholders.

## Conclusions

This study provided evidence suggesting that the two remote monitoring platforms we evaluated were useful, user-friendly, and well-received by users with no significant difference in the users' experience between the two platforms. Further research is required to support our findings and endorse if the two follow-up approaches can be used for other post-hospitalization clientele and can be considered for use even in a post-pandemic era. Finally, to maximize efficiency, improve usability, and achieve results that are even better than those recorded here, the areas for improvement and the issues identified need to be considered in a patient-centered manner.

## Data Availability Statement

The raw data supporting the conclusions of this article will be made available by the authors, without undue reservation.

## Author Contributions

M-PP, KM, AT, M-ÈD, and FL developed the idea for the study and oversaw intervention development and implementation. MT and ER collected the data. KB processed, analyzed, and interpreted the data and structured and drafted the manuscript. M-PP, BL, and DL contributed to the analysis and interpretation of the data, and supervised the writing of the manuscript, and revised it. All authors contributed to the article and approved the submitted version.

## Funding

This study was funded by the Canadian Institutes of Health Research (CIHR), Strategy for Patient-Oriented Research (CIHR Funding Reference Number: VR4−172769), the Quebec Health Research Funds (FRQS) and the ministère de la Santé et des Services sociaux du Québec for the Senior Career Award given to M-PP, as well as the Mentorship Chair in Innovative Clinical Trials for HIV Care held by BL, who was also supported by a career award LE-250 from the ministère de la Santé et des Services sociaux du Québec for researchers in Family Medicine. The CareSimple Platform was provided pro-bono to the CHUM for COVID-19 remote patient monitoring by Tactio Health Group.

## Conflict of Interest

The authors declare that the research was conducted in the absence of any commercial or financial relationships that could be construed as a potential conflict of interest.

## Publisher's Note

All claims expressed in this article are solely those of the authors and do not necessarily represent those of their affiliated organizations, or those of the publisher, the editors and the reviewers. Any product that may be evaluated in this article, or claim that may be made by its manufacturer, is not guaranteed or endorsed by the publisher.

## Footnotes

^1^Available online at: https://www.inspq.qc.ca/covid-19/donnees (accessed October 25, 2021).

^2^Available online at: https://msss.gouv.qc.ca/professionnels/maladies-infectieuses/coronavirus-2019-ncov/ (accessed October 25, 2021).

^3^Available online at: https://www.chumontreal.qc.ca/crchum/nouvelles/le-programme-techno-covid-partenariat-un-programme-de-recherche-en-soutien-aux (accessed October 25, 2021).

^4^Available online at: https://CareSimplehealth.com/ (accessed October 25, 2021).

^5^Available online at: https://redcap.chumontreal.qc.ca/redcap/ (accessed October 25, 2021).

## Abbreviations

CHUM: Université de Montréal Hospital Center
CRCHUM: Université de Montréal Hospital Research Center
CNFO: Center of Network Flow Optimization
RPM: Remote Patient Monitoring
WHO: World Health Organisation.
